# Infrared Thermographic Evaluation of Temperature Modifications Induced during Implant Site Preparation with Steel vs. Zirconia Implant Drill

**DOI:** 10.3390/jcm9010148

**Published:** 2020-01-05

**Authors:** Antonio Scarano, Felice Lorusso, Sammy Noumbissi

**Affiliations:** 1 Department of Medical, Oral and Biotechnological Sciences and CeSI-Met, University of Chieti-Pescara, Via Dei Vestini, 31, 66100 Chieti, Italy; 2 Zirconia Implant Research Group (Z.I.R.G), International Academy of Ceramic Implantology, Silver Spring, MD 20910, USA; sammy@iaoci.com; 3 Department of Oral Implantology, Dental Research Division, College Ingà, UNINGÁ, Cachoeiro de Itapemirim 29312, Brazil; 4 Department of Medical, Oral and Biotechnological Sciences, University of Chieti-Pescara, Via dei Vestini, 31, 66100 Chieti, Italy; drlorussofelice@gmail.com

**Keywords:** zirconia drill, dental implant, drilling, heat generation, osseointegration, infrared thermography

## Abstract

Background: The heat produced during implant site osteotomy can potentially interfere with and influence the osseointegration process of a dental implant. The objective of this in vitro investigation was to measure the temperature changes during simulated osteotomies in bovine rib bone. The measurements were made at the apical area of the osteotomies with steel implant drills compared to zirconia implant drills. Methods: Steel cylindrical drills (2 mm) and zirconia cylindrical drills (2 mm) were evaluated in vitro using bovine rib bone for a total of five groups based on the number of osteotomies performed with each drill: 10, 20, 40, 90, or 120 osteotomies. Bone and apical drill temperatures were measured by means of infrared thermography. The drilling time for each osteotomy was measured for each preparation. Results: Statistically significant differences were found in the temperature measurements in the bone and apical portion of the drills between the study groups (*p* < 0.05). A statistically significant difference was observed for drilling time preparation between steel cylindrical drill (2 mm) and zirconia cylindrical drills (2 mm) (*p* < 0.01). Conclusions: The drill material has an impact on the temperature changes that occur at its apical portion during bone preparation for implant placement.

## 1. Introduction

Oral implant rehabilitation is a highly predictable procedure characterized by 10-year success rates of over 97% [1,2,3]. Bone healing around the implant surface is influenced by different factors such as heat generation during implant site preparation [4,5], insertion torque, micro and macro implant surface characteristics, and quality of bone [6,7].

Bone healing around fixtures is a biological phenomenon with the proliferation and differentiation of pre-osteoblasts into osteoblasts, the production and mineralization of osteoid matrix followed by the organization of the bone–implant interface [8]. These complex biological phenomena allow the dental implant to achieve osseointegration [8].

The implant bed preparation is very important and can negatively influence the bone healing process [1]. During implant site preparation, the amount of heat generated and transferred between the drill and the bone depends on multiple factors such as the drill rotation speed [9], number of drills used [10], their design [5], and diameter [11], which have all been widely investigated.

Other important factors in heat generation are the cooling mechanisms, applied protocols [12] and the forces applied during site preparation [13].

If the implant drill is allowed to heat up above a certain temperature during bone preparation, it can cause bone necrosis. The chances of bone necrosis grow exponentially with the increase of temperature and the duration of the thermal injury [14]. After implant preparation, there is an initial resorption of bone that is followed by newly formed vital bone during a two-week period [15]. The temperature generated during the surgical preparation for implant placement is generally recorded in the region of 56 °C, as a matter of fact at 56 °C the alkaline phosphatase is denatured and bone healing is slowed down [16]. Thermal damage induced by bone drilling represents a critical factor for early implant failure [17]. Bone necrosis as a result of elevated temperatures has been previously reported in the literature [18].

Implant site preparation can cause not only a temperature increase in the bone but also mechanical damage such as microcracks in the bone involved [19]. The preservation of bone cell vitality is an important prerequisite for this the healing and maturation process, and to establish a stable bone-to-implant contact [20]. Today in the clinical setting, the three main techniques used for implant site preparation are:

1. sequential drilling with increasing drill diameter [21],
2. piezosurgery implant preparation [22,23], and
3. subsonic implant preparation [24].

Additionally, different drill materials have been proposed as such steel, zirconia, and nitride titanium.

Zirconium dioxide or zirconia is a good material used in implantology for its biocompatibility as well as physical and aesthetic properties [25,26]. In clinical practice, it zirconia is used for implant abutments and superstructures because of its durability, strength, corrosion resistance, and response to disinfection and sterilizing agents [25].

The aim of this study was to compare the temperature changes during implant bed preparation using a steel vs. a zirconia implant drill of the same cylindrical shape.

## 2. Materials and Methods

Steel and zirconia implant drills were evaluated in bovine rib bone. Twenty-four bovine ribs were cleaned and removed of all soft tissue residues, then immersed in a physiological saline water below to simulate body temperature. The inferior half of the bone was submerged in a temperature-controlled saline bath (37.0 °C). Care was taken to select samples where the bone was as homogeneous as possible, and the cortical layer was of a similar thickness for all implant sites. Each bovine rib was then secured to the aluminum base plate with adjustable clamps. Site preparation began when the internal temperature of the bone, as measured by the infrared thermography, reached the bath temperature of 37.0 ± 0.1 °C. Saline solution at room temperature was used to irrigate the site and was maintained continuously throughout drilling at a rate of 40 mL/min at room temperature. Thermal measurements were performed in a climate-controlled room (temperature: 23–24 °C, relative humidity: 50% ± 5%, and no direct ventilation on the bone).

**Figure 1:**
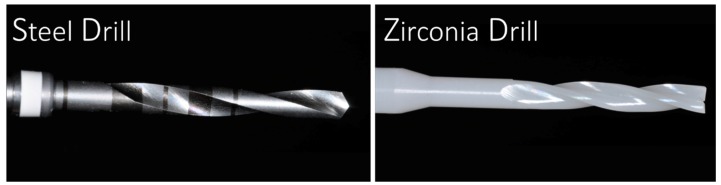
Steel cylindrical 2 mm diameter drill and zirconia cylindrical 2 mm diameter drill used for the investigation.

**Figure 2:**
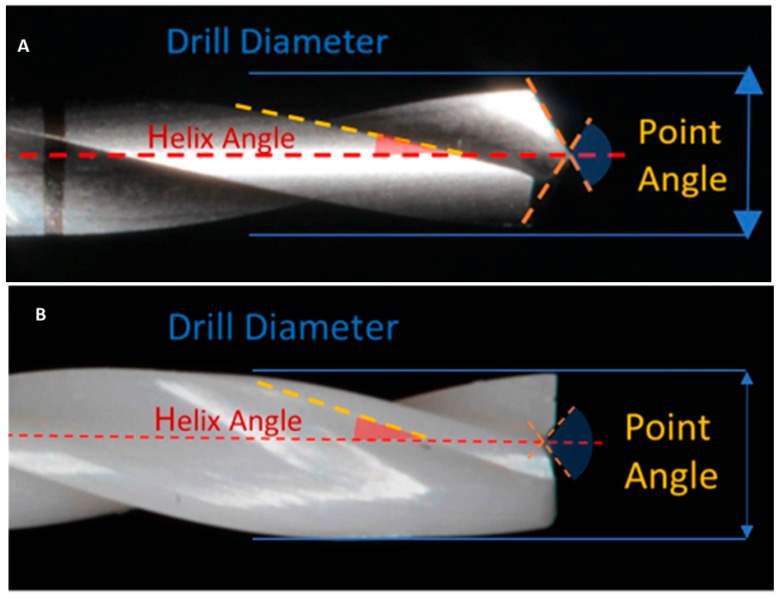
(**A**) Steel cylindrical 2 mm diameter drill with negative point angle ~110°, helix angle ~25°. (**B**) Zirconia cylindrical 2 mm diameter drill with positive point angle ~120°, helix angle ~20°.

The steel and zirconia drills evaluated were cylindrical (2 mm) with a double twist system. Twenty sets of new steel drills (Sweden Martina, Padova, Italy) and twenty zirconia drills (SAFE Implant, Malaysia) were evaluated for each system (Figure 1 and Figure 2A,B).

The drills were used sequentially for up to 120 osteotomies and the experimental data was grouped by the number of osteotomies done. The experimental data were grouped according to the number of osteotomies performed for a total of five wear groups: Group 1, 10 osteotomies; Group 2, 20 osteotomies; Group 3, 40 osteotomies; Group 4, 90 osteotomies; and Group 5, 120 osteotomies.

**Figure 3:**
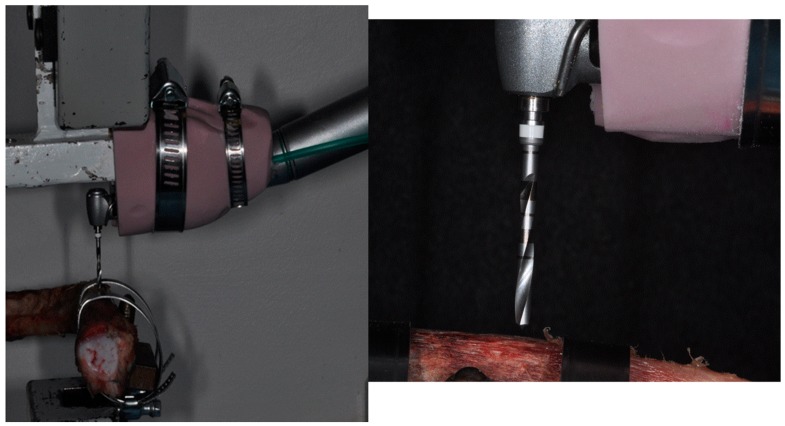
A conventional dental handpiece with a physio-dispenser mounted on a universal testing machine, before the test.

All drilling was prepared to 10 mm depth at a speed of 800 rev/min under abundant external irrigation with saline solution. The rotational speed of 800 rpm was used for easy comparison with previous work [27]. A 20:1 implant handpiece with a physio-dispenser (Vario-Surgery NSK, Tochigi, Japan) was mounted on a universal testing machine, so that there was a constant drill load (Figure 3). Continuous drilling was performed with a Lloyd 30K universal testing machine (Lloyd Instruments Ltd., Segensworth, UK), with constant load applied during implant site preparation, which was 2 kg during the entire implant preparation, and a constant torque of 40 N/cm. Moreover, the drilling depth parameter of 10 mm was electronically set for both drill groups using the Lloyd 30K universal testing machine to ensure the reliability and repeatability of the experiment. During implant preparation, the bone rib was always in a thermostat-controlled saline bath leaving 3 mm of bone emerged out of solution. The drills were not sterilized or disinfected, only cleaned. The time taken to perform the osteotomy was recorded and expressed in seconds.

**Figure 4:**
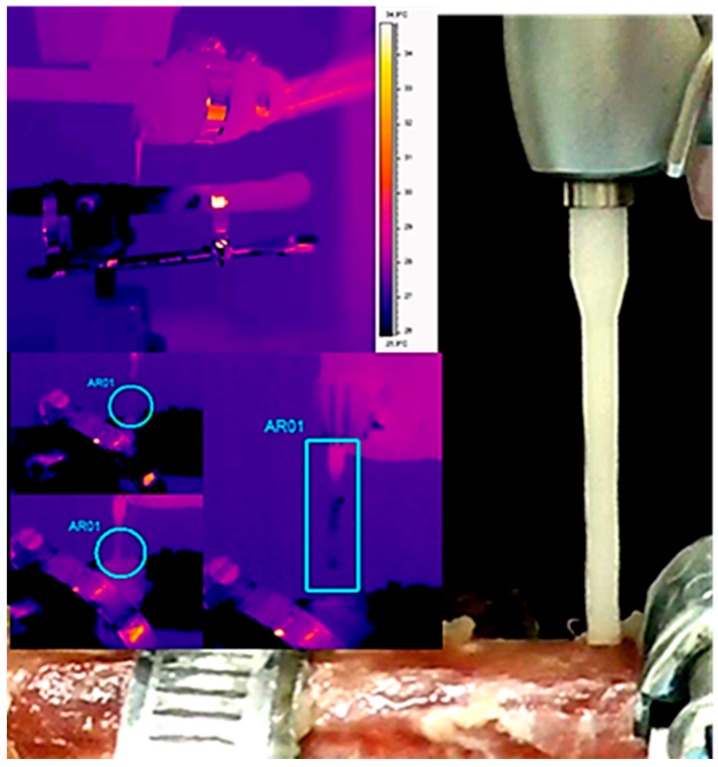
Infrared thermography temperature evaluation of the zirconia cylindrical drill (2 mm).

**Figure 5:**
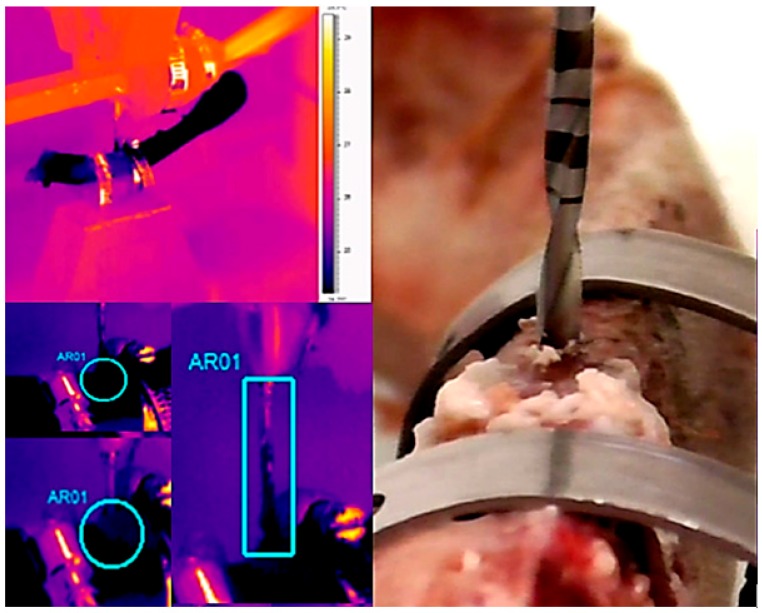
Infrared thermography temperature evaluation of the steel cylindrical drill (2 mm).

Thermal image series during implant site preparation were obtained using a 14-bit digital infrared camera (FLIR SC3000 QWIP, FLIR Systems, Danderyd, Sweden). The acquisition parameters were: 320 × 240 focal plane array; 8–9 µm spectral range; 0.02 K noise equivalent temperature differences (NETD); 50 Hz sampling rate; optics: germanium lens; f 20; and f/1.5). Images were acquired at a rate of 10 images per second and subsequently re-aligned using an edge-detection-based method implemented with in-house software. A video was performed, and the photos were extrapolated via dedicated software (FLIR Reporter, Danderyd, Sweden). The infrared thermographic system was positioned at a focal distance of 1 m from the specimens. The implant bed was positioned in a way that it was perpendicular to the surface from which the thermal image system measured any observed temperature change. To avoid the interference of water with infrared radiation emitted from the specimens, a plastic screen was applied that protected the flat bone surface of interest from the irrigant. Temperature changes in cortical bone during implant bed preparation were determined using these images (Figure 4 and Figure 5). The temperature changes in the apical portion of the drill were determined using thermal image after finishing the preparation of the implant bed and removing the drill from the bone (Figure 4 and Figure 5).

### Statistical Evaluation

A power analysis was performed using clinical software for determining the number of drills needed to achieve statistical significance for quantitative analyses of temperature. A calculation model was adopted for dichotomous variables (yes/no effect) by putting the effect incidence designed to discern the reasons, 85% for zirconia drill and 20% steel drill with alpha = 0.05 and power = 90%. The optimal number of samples for analysis was 20 drills per group. The data were analyzed with the Shapiro–Wilk test of normality and *t*-test for zirconia and steel drills samples.

The differences in temperature between the five osteotomies groups were analyzed using Welch correction ANOVA followed by Games–Howell post hoc test. Differences will be considered statistically significant at a value of *p* < 0.05.

## 3. Results

The mean temperature produced in cortical bone during implant preparation are shown in Table 1. The rise in temperature was statistically higher when over 20 osteotomies were made for both groups (*p* < 0.05).

**Figure 6:**
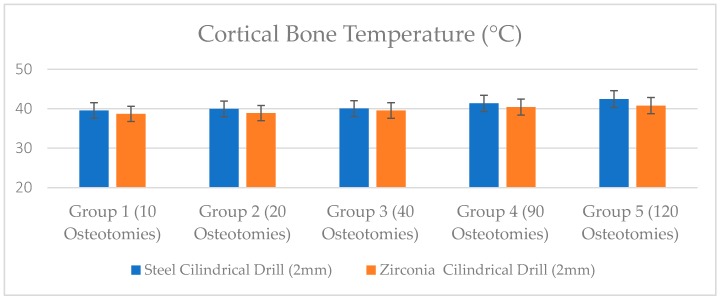
Bone temperature after site preparation with steel and zirconia drills.

No statistical difference was detected in the group 1 (*p* = 0.54). The zirconia groups showed statistically lower bone temperature compared to steel drills in Group 2, Group 3, and Group 4. After 120 osteotomies, the steel group showed a bone temperature of 42.45 ± 1.70 °C, compared to the zirconia drills which reported average values of 40.80 ± 0.85 °C (Table 1, Figure 6).

At 120 osteotomies, the mean temperature produced in the apical portion of the drill during implant preparation was 42.15 ± 1.14 °C for the steel drill and 40.62 ± 1.00 °C for the zirconia drill (Table 2).

A statistical difference in the apical temperature of the drill was present in all groups (*p* < 0.05).

The statistical difference between groups increased as the number of osteotomies increased (*p* < 0.01) (Table 2). A statistical difference was detected in the time necessary to perform the osteotomy in all groups (Table 3).

## 4. Discussion

The most interesting finding of the present study is that there was a statistically significant temperature increase and drilling time in the implant bed sites prepared with steel drills. The temperature difference between the steel and the zirconia drill was 1.5 °C. Within the limitations of this study the recorded temperature differences are not critical the health of preimplant bone. However, this difference has no clinical relevance if interpreted as an absolute value. In fact, these results are influenced by the force applied to the drill and feed rates. Inappropriate pressure during drilling may cause higher bone temperatures, which can further have an influence on the health of the peri-implant bone [28]. Moreover, the implant bed [29] preparation is complex, and the amount of pressure is influenced by multiple factors such as rotation speeds [29,30] and feed rates [31,32]. In clinical practice, it is impossible to control the pressure and feed rates of the drill. For these reasons, it can be hypothesized that in clinical practice the temperature is superior to that observed in the present study. In fact, many factors that can influence the heat generation during the implant bed preparation including drilling speed [33,34], drilling depth [35], drill geometry [36,37], sharpness of the cutting tool [38], use of internal or external irrigation [39], use of graduated versus one-step drilling [40], intermittent versus continuous drilling and drill material [41] are controllable in clinical proactive, while the pressure applied to the drill [34] is not controllable.

In the zirconia group, the outline of the implant bed was well defined even after 120 osteotomies. The temperature increase observed in the implant bed sites with the zirconia drill was probably due to their great resistance to wear. Furthermore, zirconia is also known to be a good thermal insulator. The use of zirconia material is interesting because it has conductive abilities in the bone tissue, which are almost equivalent to those of titanium implants. Moreover, zirconia drills induce less damage during implant bed preparation and advantageous for bone healing [42]. Zirconia drills, when used for implant bed preparation positively influence bone healing compared to stainless steel drills [42]. We found the generation of friction heat during osteotomies for implant preparation to be influenced by the drill material especially when we prepare implant sites in dense cortical bone. In the present research, we chose bovine ribs, which are almost similar to the human mandibular bone in terms of density and ratio between cancellous and cortical bone [14], and this model has been used by many authors.

A study showed that stainless steel and zirconia drills could be used up to 50 times without showing severe signs of wear and deformation [43].

Some studies have demonstrated that heat generation during implant bed preparation plays a significant role in implant failure [17,44]. In fact, heating of the bone induces bone devascularization, loss of vitality of the periosteum, and a denaturation of alkaline phosphatase [45].

Previous research by the current authors has used a thermocouple to measure the temperature change induced during implant site preparation in a bovine rib model [20]. A testing model was subsequently developed to visualize the temperature changes during implant site preparation under saline irrigation. A study that used external irrigation during drilling of bovine bone showed that the temperature increases with the thermocouple were significantly higher in the cortical bone, and increased when increasing the number of times of drills were used [20]. In another study, the authors used thermocouples, which provided information only about thermal changes in the area close to the drill [46,47,48]. These studies concluded that irrigation is more critical to the control of temperature elevation than the material of the drill. Furthermore, a recent research concluded that cooler irrigating solutions can confer benefits in the preparation of the implant bed by eliminating several factors that may cause bone overheating [49]. The thermocouple is fixed to the bone and, therefore, has the disadvantage of not being able to intercept the changes in temperature in the rotating drill itself.

In the present research, we used infrared thermography (IRT) evaluation because this method of measuring heat provided information about the changes in temperature in the rotating drill itself. The use of IRT for evaluating the change in temperature during implant bed preparation has the advantage of measuring the temperature of the drill but without providing information about the changes in temperature deep in the implant bed. The disadvantage of IRT is that it allows only surface temperature to be evaluated.

IRT is a well-known technique to measure infrared energy emitted from an object which it converts it into a radiometric thermal image and displays the image of surface temperature distribution. This technique is extensively used in other medical fields for evaluating the thermal distribution of a body without any contact between the body and the sensors. It is used for evaluating cutaneous temperature distribution, cutaneous blood perfusion [50], to detect varicocele [51], diabetic neuropathy [52], brain imaging (thermoencephaloscopy) [53], and breast cancer detection [54].

This technique was used for evaluating the temperature of bone during implant bed preparation in 2011 [46]. This method is now also used in the dental implantology field for measuring bone thermal changes [27,55].

The study model used in this work allowed us to evaluate the temperature in the cortical bone and in the apical portion of the drills and to demonstrate that these temperature modifications were correlated to the drill geometry. The results of the present study demonstrate that the material of the drill is also an important factor in heat generation during implant site preparation. In the present study, no consideration was given to either the influence of disinfection and sterilization or the extent of drill use. Although many factors may play a role in drill cutting efficiency and bone temperature, it is their net effect that has a clinical relevance. A review on bone drilling has investigated the methods for reducing thermal osteonecrosis [55].

In fact, the implant failure rate for osseointegration is influenced by many factors and one of them is thermal damage in bone tissue, that is influenced by drilling speed, feed rate, cooling, drill diameter, drill point angle, drill material and wear, drilling depth, pre-drilling, drill geometry, and bone cortical thickness [56]. To reduce heat generation during bone drilling, drill design, drilling parameters, coolant delivery and temperature have been studied. Currently these issues have not yet been clarified because it is difficult to define the variable most responsible for bone heating during drilling. It is difficult to measure the bone temperature during drilling, because bone is a composite of organic and inorganic components and has anisotropic behavior [57]. Moreover, the medullary cavity is a gelatinous structure, contributing to thermal dissipation.

For these reasons, it can be hypothesized that in clinical practice the temperature is higher than that observed in the present study. An in vitro study is a simple way to test some hypotheses. The methods used in the present study could provide valuable information for implantology, but it represents a simplification of the clinical reality. The outcomes of the present study were insufficient for precise and conclusive results. Different variables lead to experimental errors. In fact, the bone is a complex anisotropic and mineralized connective tissue with organic and inorganic components. Moreover, there are great individual differences, and the densities of the ribs used in this study were inhomogeneous for bone cortical thickness, even if the specimens were drilled in the same position. In this study, we used a very different in vitro model from vital bone, while in the clinical practice, the drilling is performed in bone with blood flow response to surgical trauma [58]. Finally, the drill shapes used were very similar, but not identical. This aspect can be considered negligible in consideration of the low friction forces related with the reduced diameter and the high penetrating capability of the drills investigated in this study but it could be critical in the case of increased drill diameter.

## 5. Conclusions

In conclusion, drill material plays an important role in thermal changes during implant bed preparation. Implant site preparation by zirconia drills could represent a useful tool for heat control during bone osteotomy in the clinical practice.

## Figures and Tables

**Table 1:** Summary of the cortical bone temperature after drilling site preparation (mean ± standard deviation).

Bone Temperature (°C)	Group 1 (10 Osteotomies)	Group 2 (20 Osteotomies)	Group 3 (40 Osteotomies)	Group 4 (90 Osteotomies)	Group 5 (120 Osteotomies)
Steel Cylindrical Drill (2 mm)	39.55 ± 0.98	39.97 ± 0.92	40.06 ± 1.26	41.37 ± 1.81	42.45 ± 1.70
Zirconia Cylindrical Drill (2 mm)	38.70 ± 0.83	38.9 ± 1.36	39.55 ± 1.79	40.43 ± 1.82	40.80 ± 0.85
*p* Value	*p* = 0.54	*p* = 0.25	*p* = 0.031 (*)	*p* = 0.0033 (**)	*p* = 0.0004 (**)

* *p* = 0.05 ** *p* = 0.01.

**Table 2:** Summary of the apical drill temperature after bone site preparation (mean ± standard deviation).

Apical Drill Temperature (°C)	Group 1 (10 Osteotomies)	Group 2 (20 Osteotomies)	Group 3 (40 Osteotomies)	Group 4 (90 Osteotomies)	Group 5 (120 Osteotomies)
Steel Cylindrical Drill (2 mm)	40.51 ± 0.88	40.63 ± 0.97	41.66 ± 0.55	41.96 ± 1.51	42.15 ± 1.14
Zirconia Cylindrical Drill (2 mm)	39.68 ± 1.10	39.75 ± 0.89	40.14 ± 1.01	40.20 ± 0.85	40.62 ± 1.00
*p* Value	*p* = 0.33	*p* = 0.025 (*)	*p* = 0.028 (*)	*p* = 0.0003 (**)	*p* = 0.0001 (**)

* *p* = 0.05 ** *p* = 0.01.

**Table 3:** Summary of the drilling time (in seconds) preparation calculated for zirconia and steel groups (mean ± standard deviation).

Drilling Time (Sec)	Group 1 (10 Osteotomies)	Group 2 (20 Osteotomies)	Group 3 (40 Osteotomies)	Group 4 (90 Osteotomies)	Group 5 (120 Osteotomies)
Steel Cylindrical Drill (2 mm diameter)	11.05 ± 0.91	11.08 ± 1.09	11.56 ± 0.56	12.15 ± 0.70	12.88 ± 1.34
Zirconia Cylindrical Drill (2 mm diameter)	9.62 ± 0.75	9.79 ± 0.52	10.02 ± 0.62	10.44 ± 0.62	10.53 ± 1.09
*p* Value	*p* = 0.0067 (**)	*p* = 0.0003 (**)	*p* = 0.0001 (**)	*p* = 0.00003 (**)	*p* = 0.000001 (**)

* *p* = 0.05 ** *p* = 0.01.
